# Eph/Ephrin-mediated stimulation of human bone marrow mesenchymal stromal cells correlates with changes in cell adherence and increased cell death

**DOI:** 10.1186/s13287-018-0912-3

**Published:** 2018-06-26

**Authors:** David Alfaro, Agustín G. Zapata

**Affiliations:** Department of Cell Biology, Faculty of Biology, Complutense University of Madrid, C/ José Antonio Novais, 12, CP 28040 Madrid, Spain

**Keywords:** Eph/Ephrin blockade and activation, MSC survival and morphology

## Abstract

**Background:**

Mesenchymal stromal cells (MSC) are components of connective tissues and, in vitro, cell entities characterized by cell adhesion and immunophenotyping, although specific markers for their identification are lacking. Currently, MSC derived from either human bone marrow (BM-MSC) or adipose tissue (Ad-MSC) are considered the main sources of MSC for cell therapy. Eph receptors and their ligands, Ephrins, are molecules involved in cell adhesion and migration in several tissues and organs. In the current study, we analyze the pattern of Eph/Ephrin expression in MSC and evaluate the effects of blockade and stimulation of these receptor/ligand pairs on their biology.

**Methods:**

Eph/Ephrin expression was analyzed in both BM-MSC and Ad-MSC by qRT-PCR. Then, we supplied BM-MSC cultures with either blocking or activating compounds to evaluate their effects on MSC proliferation, survival, and cell cycle by FACS. Changes in cytoskeleton and integrin α5β1 expression were studied in stimulated BM-MSC by immunofluorescence microscopy and FACS, respectively.

**Results:**

Higher numbers of Eph/Ephrin transcripts occurred in BM-MSC than in Ad-MSC. In addition, the blocking of Eph/Ephrin signaling correlated with decreased numbers of BM-MSC due to increased proportions of apoptotic cells in the cultures but without variations in the cycling cells. Unexpectedly, activation of Eph/Ephrin signaling by clustered Eph/Ephrin fusion proteins also resulted in increased proportions of apoptotic MSC. In this case, MSC underwent important morphological changes, associated with altered cytoskeleton and integrin α5β1 expression, which did not occur under the blocking conditions.

**Conclusions:**

Taken together, these results suggest that Eph/Ephrin activation affects cell survival through alterations in cell attachment to culture plates, affecting the biology of BM-MSC.

**Electronic supplementary material:**

The online version of this article (10.1186/s13287-018-0912-3) contains supplementary material, which is available to authorized users.

## Background

Mesenchymal stromal cells (MSC) were described initially in the bone marrow and later in the connective tissue of almost all organs [1]. Over the past years, MSC identified as components of the bone marrow hematopoietic niches have been found to exhibit important immunomodulatory properties and are extensively being used in cell therapy [2]. Nevertheless, due to the contradictory clinical results obtained using these cells, it is important to improve their therapeutic efficiency, an issue clearly associated with a better knowledge of their biology.

Eph kinases represent the largest family of tyrosine kinase receptors and bind to membrane-bound ligands, the Ephrins. Eph receptors are divided into family A (10 members), which preferentially, but not exclusively, bind EphrinA ligands (6 members), and family B (6 members), which bind EphrinB (3 members) [3]. Eph and Ephrins provide positional information for cells and regulate cell-to-cell contacts, cell migration, attraction-repulsion processes, cell survival, and differentiation [4, 5]. MSC derived from the stromal fraction of bone marrow (BM-MSC) or umbilical cord blood express Eph and Ephrins, particularly those of family B [6–10]. In addition, they are expressed in hematopoietic progenitor cells, largely in the most primitive cells [9, 11–14]. However, the role played by Eph/Ephrins in the biology of MSC is a matter of discussion.

Eph/Ephrin signaling controls adhesion between MSC and other cell components of bone marrow, favoring or blocking their mobilization and differentiation or the maintenance of hematopoietic stem precursor cells (HSPC) as well as the commitment of BM-MSC to either adipogenic or osteogenic cell lineages. EphB signaling inhibits adhesion between dental niche stem cells and MSC [15], and the presence of an EphB4-specific blocking peptide significantly decreases the adhesion of CD34^+^ HSPC seeded onto human BM-MSC [9]. These changes in cell adhesion correlate with cell mobilization of dental niche MSC [15, 16], and ectopic expression of EphrinB2 in the hematopoietic cell line 70Z/3 significantly reduces the locomotive activity of hematopoietic cells underneath a stromal cell layer [17]. Also, in-vivo intraperitoneal injection of EphA3-Fc fusion proteins enhances mobilization of murine HPSC into the peripheral blood, whereas the blocking of EphA3-EphrinA5 interactions affects the HSPC homing [14, 18]. Kwak et al. [8] recently reported that EphrinB2/EphB4 signaling regulates HSPC exit from the bone marrow but not their homing and engraftment. However, high EphB2 expression enhances homing and engraftment of human bone marrow stromal cells [19].

Furthermore, Eph/Ephrin signaling affects MSC morphology. A 3-h treatment with EphB2-Fc or EphB4-Fc, but not with EphrinB1-Fc or EphrinB2-Fc, produces roundness and decreased size of Stro-1^+^ BM-MSC [6], and EphA3^+^ CD29^+^ Sca-1^hi^ MSC isolated from tumor niche respond to an EphA3-activating monoclonal antibody (mAb) by fast contraction and apoptosis [20]. Remarkably, small, rounded MSC are prone to differentiate into adipocytes, whereas large, extended MSC differentiate rather into the osteogenic lineage [21, 22].

On the other hand, EphB2-Fc treatment increases osteogenesis, whereas blocking EphB1 or EphB4 signaling peptides inhibits it [6], and EphA5 has been described as an inhibitor of osteogenesis [23]. In addition, Eph/Ephrins, specially the EphB4/EphrinB2 pair, are involved in the maturation of primitive hematopoietic precursor cells [9, 13, 24].

The present study analyzes the expression of Eph/Ephrins in BM-MSC and adipose tissue-derived MSC (Ad-MSC) of healthy donors. We further studied the effects of blockade/stimulation of Eph/Ephrin signaling on the survival, proliferation, and differentiation, parameters studied in numerous cell types but not in BM-MSC, observing that the blockade of Eph-Ephrin interactions produces increased proportions of apoptotic MSC without significant changes in their proliferation or cell morphology. On the contrary, stimulation of both Eph and Ephrins by clustered fusion proteins correlates with important changes in the cell morphology and adhesion that result, in the most severe cases, in cell death and decreased proportions of proliferating cells supporting a correlation between increased apoptotic MSC and loss of cell adhesion.

## Methods

### MSC samples and cultures

Human MSC were obtained from either adipose tissue (Hospital Universitario Niño Jesús, Madrid) or total bone marrow (Hospital Universitario de Valladolid) from at least five healthy donors in accordance with the Declaration of Helsinki. This study was approved by the Ethical Committees of Clinical Research of both hospitals. Cells were harvested, cultured as previously described [25], and phenotypically characterized by flow cytometric analysis of the expression of a panel of cell markers recognized for defining MSC (positive for CD73, CD90, CD105, and CD166, but not for CD14, CD34, CD45, or HLA-DR). These cells were then frozen in 10% dimethyl sulfoxide (DMSO; Sigma-Aldrich, USA) in fetal bovine serum (FBS; Gibco, USA) and sent to our laboratory. After thawing, cells were cultured until around 80% confluence and then recovered by trypsinization, counted, and replated at a density of 5 × 10^3^ cells/cm^2^ to be expanded in complete culture medium of Dulbecco’s modified Eagle’s medium (DMEM; Lonza, Spain) 10% FBS (Gibco), 1% antibiotic/antimycotic (Gibco), 1% l-glutamine (Sigma-Aldrich) and 1% sodium pyruvate (Sigma-Aldrich). All the cells were incubated in a cell culture incubator (Forma Scientific, USA) at 37 °C in a humidified atmosphere containing 5% CO_2_. All experiments were performed with cells harvested between passages 4 and 7.

### Quantitative real-time polymerase chain reaction (qRT–PCR)

RNA was isolated from Ad-MSC or BM-MSC using the Total RNA Extraction kit (Biotools, Spain) according to the supplier’s instructions. Total cDNA from these cells was synthesized by a High Capacity cDNA Reverse Transcription Kit (Applied Biosystems, USA) and then used as a target in the PCR amplifications. RT-PCR was performed with specific primers for the Eph and Ephrin genes (Table 1). All PCR reactions were set in duplicate using the SYBR Green Master Mix (Applied Biosystems). Gene expression was normalized with respect to relative expression of β-actin (NM_001101.3) housekeeping gene and was calculated based on the threshold cycle values, using the 2^–ΔΔCT^ method. Amplifications and analyses were performed in a 7.900HT Fast Real-time PCR System (Genomic Centre, Complutense University of Madrid, Spain).

**Table 1:** Eph/Ephrin quantitative real-time polymerase chain reaction primers

Gene		Primer sequence
EphB1	Forward	tggctatgagcctgagaacag
	Reverse	agtgggagcagccttcag
EphB2	Forward	tgtctcagatgatgatggagga
	Reverse	ccgcatcacctggatactgt
EphB3	Forward	tgccaaggagtcccagtg
	Reverse	aggtggtacggctgttgg
EphB4	Forward	cggatcctacccgagtga
	Reverse	tgtgttcagcagggtctcttc
EphB6	Forward	tcctcgaatggcagaaaaag
	Reverse	ttctgcaaggggttattcca
ephrinB1	Forward	tcatgaaggttgggcaaga
	Reverse	cagtgttgtctgcctccttg
ephrinB2	Forward	tctttggagggcctggat
	Reverse	ccagcagaacttgcatcttg
ephrinB3	Forward	tggaactcggcgaataagag
	Reverse	cgatctgagggtacagcaca
EphA1	Forward	tggagagtgaccaggatgtg
	Reverse	tcgaatggtgaagctctggt
EphA2	Forward	ccaggcaggctacgagaa
	Reverse	ggctctcagatgcctcaaac
EphA3-L	Forward	attttggcaatgggcattta
	Reverse	atgtatgtgggtcaacataagtcc
EphA3-C	Forward	attacttccgggcagacaaa
	Reverse	ttcttggtgaagatggaggtc
EphA4	Forward	catgtcccgagtgcttgag
	Reverse	cagtccaccggataggaatc
EphA5	Forward	gcgtcctcctcagtggaa
	Reverse	cgccatattaggcttggatg
EphA6-A	Forward	tgaatttgaaacaggagatgaaac
	Reverse	cggtggctatcacgagaatc
EphA6-B	Forward	gagactgagaaccaagcctca
	Reverse	aggtcagctgctcatgttcc
EphA7	Forward	gaggatgatccagaagctgtctat
	Reverse	ggtgctgtccaccttactgg
EphA8–1	Forward	atgcactatcagaatggacagg
	Reverse	cgcatagaactggggctct
EphA8–2	Forward	acctcagctactaccgtgcag
	Reverse	actggagatcaggttcactgg
EphA10–2	Forward	catgaggtctcctcagtgcata
	Reverse	atgcaaccaatcccactagc
EphA10–3	Forward	caagtgtgccctgactacctg
	Reverse	cccacagagccaaaggag
EphrinA1-A	Forward	gaaggacacagctactactacatctcc
	Reverse	ggcctgaggactgtgagtg
EphrinA1-B	Forward	tccagcgcttcacacctt
	Reverse	ggcctgaggactgtgagaga
EphrinA2	Forward	ctccctgggcttcgagtt
	Reverse	ccgcacgtacaccttcagt
EphrinA3	Forward	actctcccccagttcacca
	Reverse	gcacctgagggttctctcc
EphrinA4-A	Forward	gaggctccaggtgtctgtct
	Reverse	ccaacaggatgggctgac
EphrinA4-B	Forward	ctacatctcggtgcccactc
	Reverse	cttgggaggactctggctct
EphrinA4-C	Forward	tctgctgcaaggagaggaac
	Reverse	tcctcctccaggggatctt
EphrinA5	Forward	cactcccttttctctaggatttga
	Reverse	gacaggaccttcttccattatctg
β-actin	Forward	ccaaccgcgagaagatga
	Reverse	ccagaggcgtacagggatag

Sequences of specific primers used to analyze the expression of Eph/Ephrin genes in human Ad-MSC and BM-MSC by qRT-PCR

### Eph/Ephrin treatment of MSC cultures

For blocking treatments, 20,000 BM-MSC were seeded in duplicates on P24 plates for 24 h before being treated for 3 or 6 days with different blocking reagents: Eph/Ephrin-Fc fusion proteins (5μg/ml; R&D Systems, USA) (see concrete molecules in each figure); UniPR129 and UniPR500 (30 μM) provided by Dr. Massimiliano Tognolini (University of Parma, Italy), and an anti-EphrinB2 mAb scFv B11 (5 μg/ml), a gift of Dr. Jorge Martínez-Torrecuadrada (CNIO, Madrid, Spain). Treatments to activate Eph/Ephrin signaling incubated culture plates with 10 μg/ml anti-hIgG Fc (Jackson ImmunoResearch, USA). The anti-hIgG Fc was diluted on sterile phosphate-buffered saline (PBS) and incubated at 37 °C for 1 h. Then it was removed and different combinations of Eph/Ephrin-Fc proteins (5 μg/ml) (see molecules used in each figure) were added and incubated for 1 h at 37 °C. To minimize possible interactions between receptors and ligands in the combined Eph-Fc plus Ephrin-Fc treatments, the fusion proteins were highly diluted in PBS (1/100) and the mixture was made just before adding to the culture plate wells. Finally, 20,000 BM-MSC were seeded in duplicate on P24 plates and treated for 3 or 6 days with the different reagents. All the treatments were diluted in complete DMEM culture medium, except UniPR molecules that were diluted in DMSO. Cells were then photographed on a Nikon Eclipse inverted microscope (Nikon, Japan) provided with a Motic camera (Motic, Hong Kong), and harvested to evaluate their viability and cell cycle (see below). In the cases in which Eph/Ephrin signaling activation produced cell aggregates, the viability of grouped cell masses versus that of BM-MSC that remained isolated in the cultures was evaluated by Annexin V staining. As an internal control, the proportions of viable BM-MSC were measured in cultures grown on Ultra-Low attachment P24 plates (BD, USA) to obtain cell aggregates emulating those obtained after Eph/Ephrin activation.

### FACS analyses: apoptosis, cell cycle and integrin expression

After 3 or 6 days of culture, treated and nontreated cells were harvested and counted after Trypan blue staining in a Neubauer chamber while the proportion of apoptotic MSC and cycling cells were determined in a FACSCalibur flow cytometer (BD Biosciences, USA) at the Cytometry and Fluorescence Microscopy Centre of Complutense University (UCM). Apoptosis was determined by staining with the DY634 Annexin V Apoptosis Detection Kit (Immunostep, Spain) according to the supplier’s instructions. Apoptotic cells, based on propidium iodide (PI) staining, were defined as Annexin V^+^-PI^−^ cells. To determine the percentage of cycling cells (S + G2/M), treated BM-MSC were incubated for 30 min with DNA Labeling Solution (Cytognos, Spain) and then analyzed by FACS. The percentage of integrin α or β chain expressing cells on treated and control cell cultures were evaluated using P1D6-I mouse IgG anti-α5 chain integrin (a gift from J. Teixidó, CIB, Madrid) and mouse IgG anti-β1 chain integrin (Sigma-Aldrich) antibodies, which were revealed with a secondary anti-mouse IgG Alexa 488 antibody (Molecular Probes, USA).

### Immunofluorescence microscopy

The organization of the BM-MSC cytoskeleton was analyzed by immunofluorescence in both control and treated cultures that underwent cell detachment. Cells were cultured and activated with Eph/Ephrin-Fc proteins in eight-well Milicell EZ slides (Miltenyi Biotec, Germany) and then were fixed and permeabilized with Cytofix/Cytoperm (BD Biosciences) for 10 min, and washed and maintained in Permwash at 4 °C. Cells were then stained with rat IgG anti-hVimentin (Sigma-Aldrich), recognized with a secondary anti-rat IgG Alexa 488 antibody (Molecular Probes), and Phalloidin TxRed (Sigma-Aldrich) to identify actin. Slides were mounted with Vectashield antifade mounting medium with DAPI (Vectorlabs, USA) to visualize nuclei. Stained cultures were studied and photographed on an Olympus CoolLED pE-300-W microscope.

### Alkaline phosphatase activity

The activity of alkaline phosphatase (ALP) was evaluated as previously described [26] through a colorimetric assay using BM-MSC cell lysates. Briefly, 20,000 cells were seeded in duplicate on P24 plates and stimulated firstly for 3 days with clustered Eph/Ephrin-Fc fusion proteins and then for 7 more days with the Osteogenic Differentiation Bulletkit (Lonza, Switzerland). The total cellular protein content of cell lysates was determined using the Bradford method (BioRad, USA), and mean values of ALP activity (optical density (OD) 405 nm) per μg total protein are represented.

### Statistical analyses

Data are presented as mean ± standard deviation. A paired Student’s *t* test was used for statistical analysis under Microsoft Excel 2010 software. Values of *p* ≤ 0.05 (*), *p* ≤ 0.01 (**), and *p* ≤ 0.005 (***) were considered to be statistically significant.

## Results

### Larger expression of Eph and Ephrins in human BM-MSC than in Ad-MSC

**Fig. 1:**
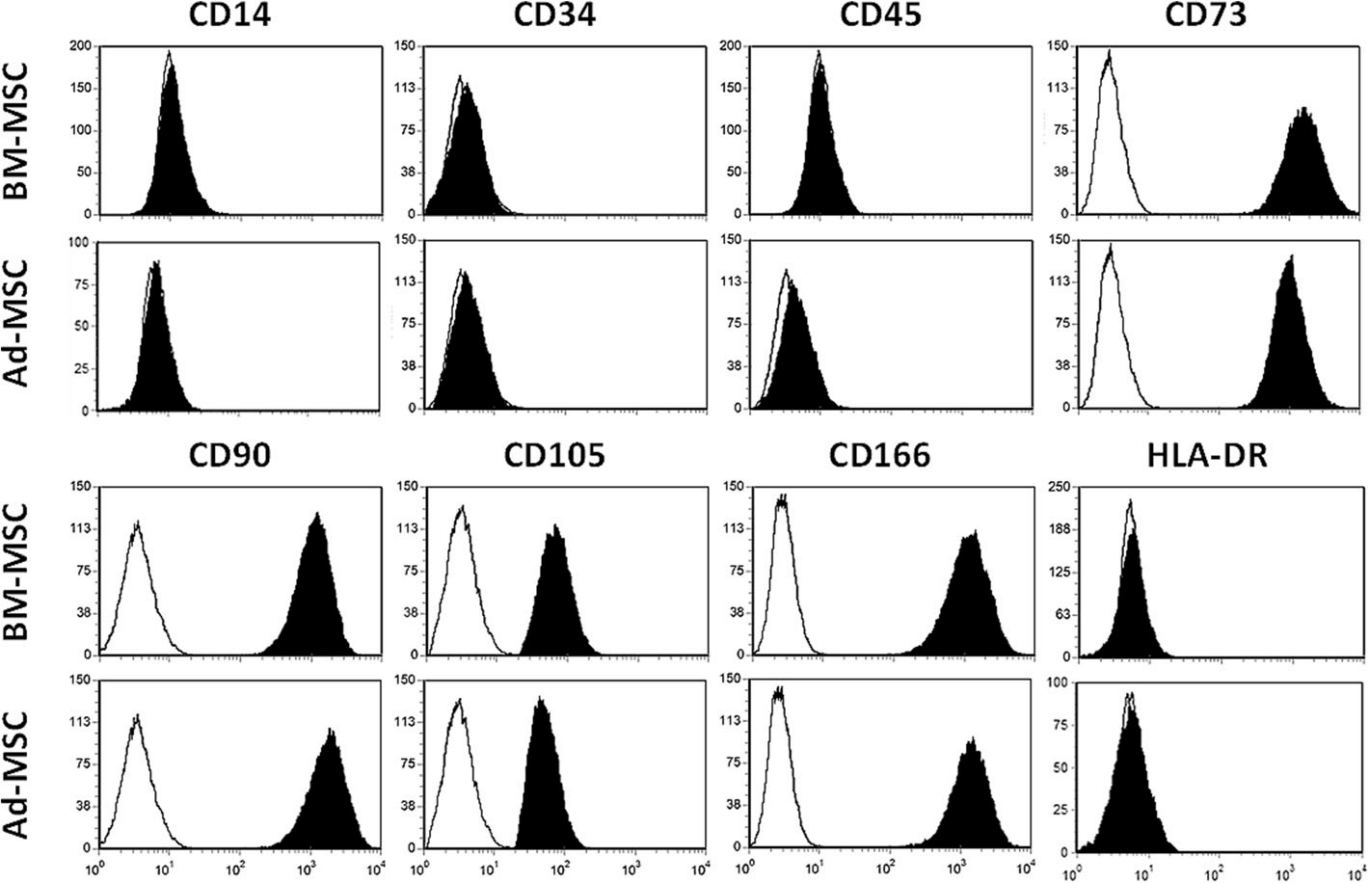
Pattern of surface marker expression of mesenchymal stromal cells (MSC) derived from either bone marrow (BM-MSC) or adipose tissue (Ad-MSC). Note the absence of differences in the expression of studied molecules between the two kinds of cells. A representative example of the profile of molecules expressed on each cell type is shown. Cells are positive for CD73, CD90, CD105, and CD166, but do not express CD14, CD34, CD45, or HLA-DR. White histograms represent background fluorescence using isotype-matched irrelevant mAbs

Both Ad-MSC and BM-MSC were isolated as previously described [25, 27] and phenotypically characterized by flow cytometry after three passages of culture. Both Ad-MSC and BM-MSC exhibited typical MSC phenotype [28], expressing CD73, CD90, CD105, and CD166, but not CD14, CD34, CD45, or HLA-DR (Fig. 1).

**Fig. 2:**
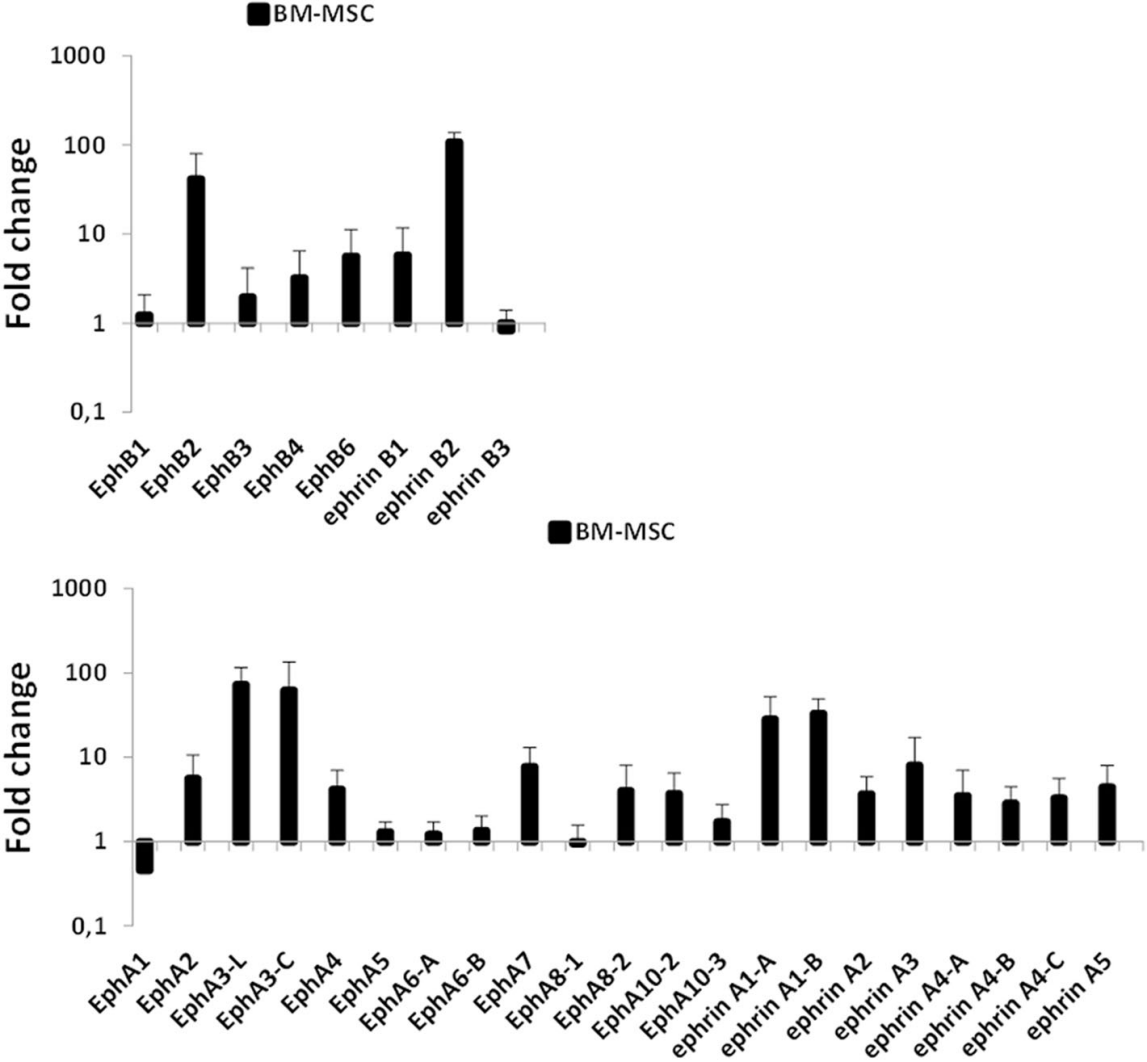
Comparative expression of Eph and Ephrin genes evaluated by qRT-PCR in Ad-MSC and bone marrow-derived mesenchymal stromal cells (BM-MSC). The figures show higher numbers of Eph/Ephrin transcripts in BM-MSC than in Ad-MSC (the reference value), particularly in the case of EphB2, EphrinB2, the two isoforms of EphA3 and EphrinA1, EphA7 and EphrinA3. Data were obtained from five different donors

Using quantitative RT-PCR, transcripts for all Eph and Ephrins were identified in both BM-MSC and Ad-MSC. Except for EphA5, EphA6, EphA8–1, EphB1, and EphrinB3, which were similarly expressed in MSC from the two sources, BM-MSC expressed a higher number of Eph and Ephrin transcripts than those isolated from adipose tissue (Ad-MSC), especially EphA3, A7, and B2, and EphrinA1, A3, and B2 (Fig. 2).

### The blockade of Eph-Ephrin signaling in BM-MSC correlated with decreased cellular growth that correlated with increased cell death and unchanged cell proliferation

**Fig. 3:**
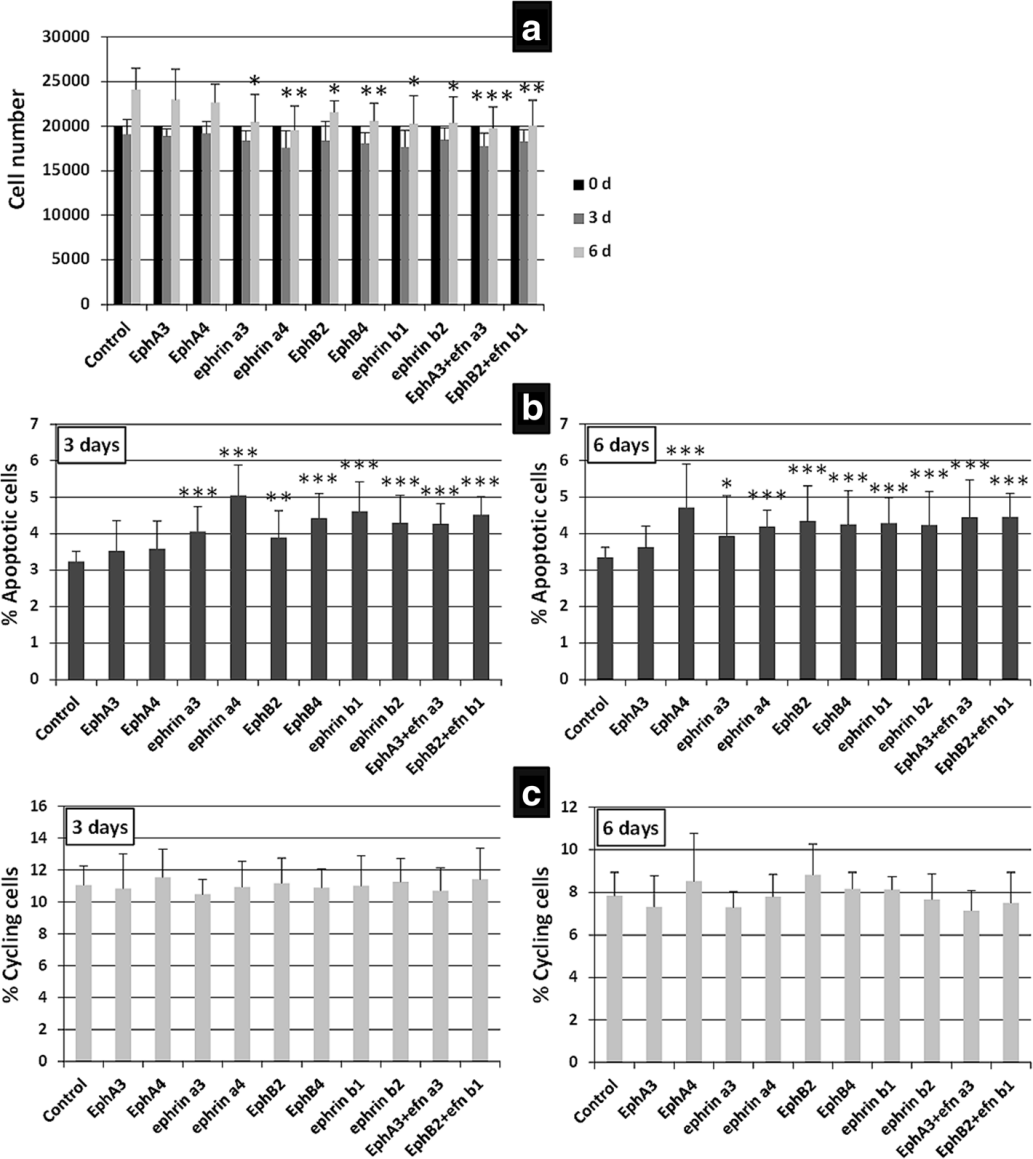
Effects of the blockade of Eph/Ephrin signaling on BM-MSC. BM-MSC cultures treated for 3 and 6 days with distinct soluble Eph/Ephrin-Fc fusion proteins that block Eph/Ephrin signaling. **a** A lower number of cells per well on day 6 under all conditions except after EphA3-Fc and EphA4-Fc treatment. **b** These decreased values correlate well with increased percentages of apoptotic MSC in treated cultures after 3 and 6 days. **c** However, no significant differences in the percentage of cycling cells at any time point are found. Data were obtained from five different donors. *p ≤ 0.05, **p ≤ 0.01, ***p ≤ 0.005, versus respective control

Because human BM-MSC expressed Eph and Ephrins more than MSC derived from adipose tissue, additional studies were performed on the BM-MSC. Firstly, we comparatively evaluated the growth kinetics of BM-MSC at 3 and 6 days after blocking Eph/Ephrin signaling with different soluble Eph-Fc and/or Ephrin-Fc fusion proteins. As expected, both treated and nontreated MSC exhibited a slight, nonsignificant decrease in the cell numbers after 3 days of culture, undergoing an increase on day 6 that was significantly lower in BM-MSC treated with either EphrinA3-Fc, EphrinA4-Fc, EphB2-Fc, EphB4-Fc, EphrinB1-Fc, EphrinB2-Fc, EphA3-Fc plus EphrinA3-Fc, or EphB2-Fc plus EphrinB1-Fc than in control, nontreated cells. On the contrary, cultures treated with either EphA3-Fc or EphA4-Fc fusion proteins did not exhibit changes in MSC numbers (Fig. 3a).

These results correlated well with the changes observed in the percentages of apoptotic BM-MSC found in the treated cells (Fig. 3b), but not with the levels of cell proliferation which did not exhibit significant variations in relation to control values of untreated BM-MSC at 3 and 6 days (Fig. 3c). At both 3 and 6 days, increased proportions of apoptotic BM-MSC were found in cultures treated with soluble EphrinA3, EphrinA4, EphB2, EphB4, EphrinB1, EphrinB2, EphA3 plus EphrinA3, and EphB2 plus EphrinB1 fusion proteins (Fig. 3b), which also exhibited reduced cellularity (Fig. 3a). Remarkably, although BM-MSC treated with EphA4-Fc proteins showed important increased apoptosis after 6 days of treatment, the values were not sufficiently high to induce a significant reduction of the cell content of these cultures.

These data support the conclusion that the blockade of Eph and/or Ephrin signaling induced increased apoptosis of BM-MSC and, consequently, reduced cellularity. However, some studies have suggested that soluble, dimeric fusion proteins, similar to those used in the current assays, although blocking Eph/Ephrin interactions expressed in different cells could activate the bound molecules [29, 30]. To clarify whether the blockade of Eph/Ephrin interactions really correlated with increased apoptotic BM-MSC, we studied the effects of other Eph/Ephrin antagonists on BM-MSC survival.

### Treatment with diverse antagonists confirms that blockade of Eph/EphrinB signaling correlates with increased proportions of apoptotic BM-MSC

Three different antagonists were tested in these assays: an anti-EphrinB2 mAb (B11) [31] blocking the interactions of EphrinB2 with all EphB, and small molecules (UniPR129 and UniPR500) largely blocking EphrinA1-EphA2 interactions, and also other ones mediated by the EphrinB1-EphB pairs [32].

**Fig. 4:**
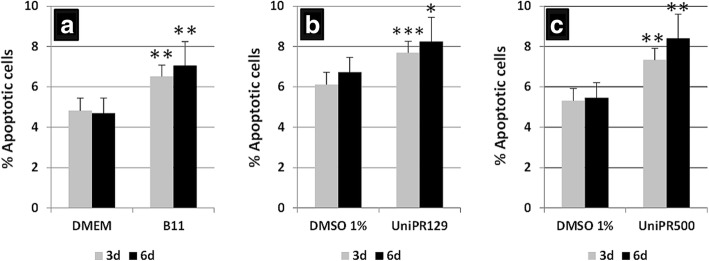
Treatments with Eph/Ephrin antagonists confirm that the blockade of Eph/Ephrin signaling results in increased cell death. To confirm the effects of soluble blocking of Eph/Ephrin-Fc fusion proteins on BM-MSC, cultures were established for 3 (gray columns) and 6 days (black columns) with different Eph/Ephrin antagonists. **a** At both time points, a blocking anti-EphrinB2 antibody, B11, produces higher proportions of apoptotic MSC than control ones supplied with Dulbecco’s modified Eagle’s medium (DMEM). **b,c** Likewise, small Eph/Ephrin blocking molecules, UniPR129 and UniPR500, affect MSC viability increasing the percentage of apoptotic BM-MSC 3 and 6 days after treatment as compared with control, dimethyl sulfoxide (DMSO)-treated cultures. Data were obtained from five different donors. *p ≤ 0.05, **p ≤ 0.01, ***p ≤ 0.005, versus respective control

Firstly, we observed that the continuous supply of 5 μg/ml mAb B11, which competes for the binding of EphrinB2 to EphB, to BM-MSC cultures for 3 or 6 days induced significantly increased proportions of apoptotic MSC (Fig. 4a). In addition, at 30 μM both UniPR129 (Fig. 4b) and UniPR500 (Fig. 4c) induced increased cell death of BM-MSC at 3 as well as after 6 days of treatment compared with proportions of apoptotic MSC treated with 1% DMSO.

### Treatment of BM-MSC with diverse clustered fusion proteins that activate Eph/Ephrin signaling also results in increased proportions of apoptotic cells

**Fig. 5:**
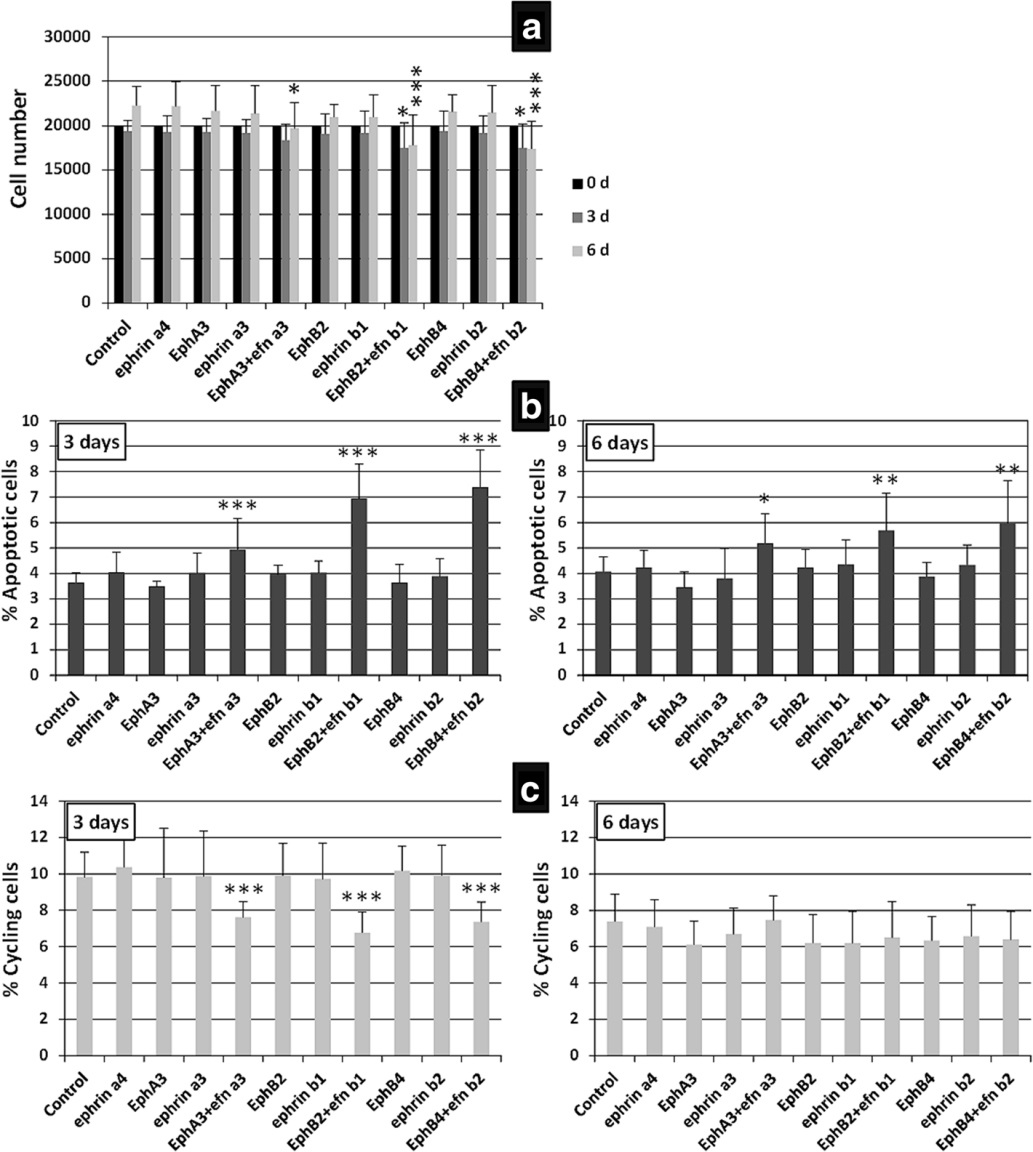
Some activating Eph/Ephrin–Fc fusion proteins alter MSC viability and proliferation. BM-MSC cultures were treated for 3 and 6 days with clustered solid-phase Eph/Ephrin-Fc fusion proteins to activate their signaling pathway. Interestingly, when MSC were double-activated through forward and reverse signals with EphA3 plus EphrinA3 (EphA3 + efnA3), EphB2 plus EphrinB1 (EphB2 + efnB1), or EphB4 plus EphrinB2 (EphB4 + efnB2), the number of cultured cells diminished (**a**), in correlation with increased percentages of apoptotic cells (**b**), and reduced cycling cells on day 3, but not on day 6 (**c**). Data were obtained from five different donors. *p ≤ 0.05, **p ≤ 0.01, ***p ≤ 0.005, versus respective control

It is generally assumed that clustered Eph or Ephrins activate Eph/Ephrin signaling pathways [33]. Therefore, we evaluated the cell content, survival, and proliferation of BM-MSC cultured for 3 or 6 days with clustered Eph-Fc and/or Ephrin-Fc fusion proteins. At 3 days of culture, the numbers of BM-MSC significantly decreased only after treatment with either EphB2-Fc plus EphrinB1-Fc or EphB4-Fc plus EphrinB2 fusion proteins, but not after supplying other individual Eph or Ephrins. At 6 days, the reduced cellularity in these cultures was even larger and also occurred in those treated with EphA3-Fc plus EphrinA3-Fc proteins (Fig. 5a). These decreased numbers of BM-MSC coincided with significantly increased proportions of apoptotic cells found in treated cultures with the same fusion proteins after 3 and 6 days (Fig. 5b). In addition, decreased percentages of cycling cells in the cultures treated with either EphA3-Fc plus EphrinA3-Fc, EphB2-Fc plus EphrinB1-Fc, and EphB4-Fc plus EphrinB2-Fc (Fig. 5c) contributed to the observed reduced cellularity (Fig. 5a). However, at 6 days no changes occurred in the proportions of cycling cells treated with any fusion protein (Fig. 5c).

### Increased apoptotic proportions of BM-MSC observed after treatment with some clustered Eph/Ephrin fusion proteins correlated well with morphological changes in the BM-MSC colonies that could favor cell detachment

**Fig. 6:**
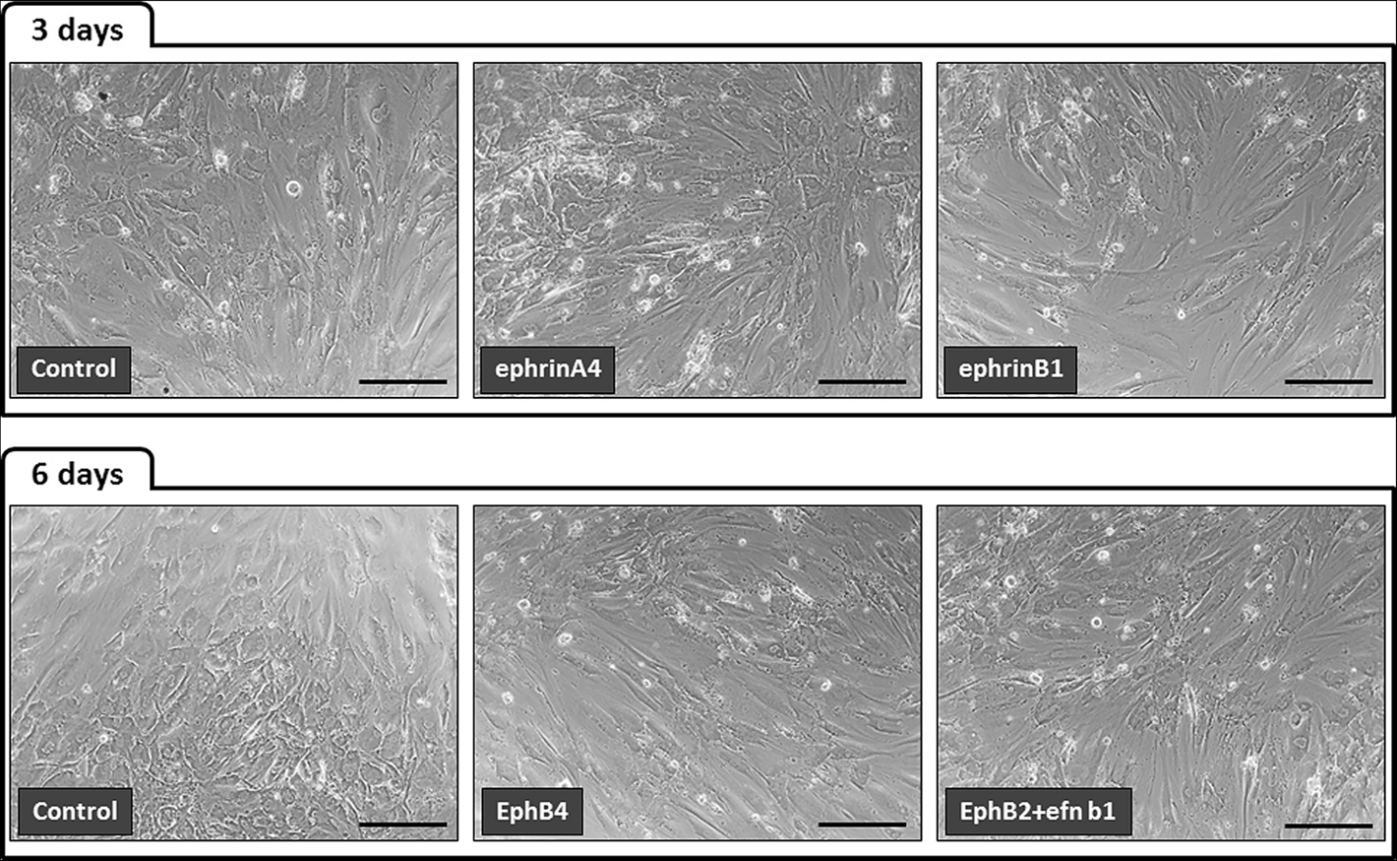
The blockade of Eph/Ephrin signaling with soluble fusion proteins does not alter BM-MSC morphology. All cell cultures treated with soluble Eph/Ephrin-Fc fusion proteins exhibit the typical elongated cell morphology of MSC without remarkable differences between treated and control, nontreated cultures. The figure shows representative pictures of BM-MSC supporting several treatments. Scale bars = 200 μm

**Fig. 7:**
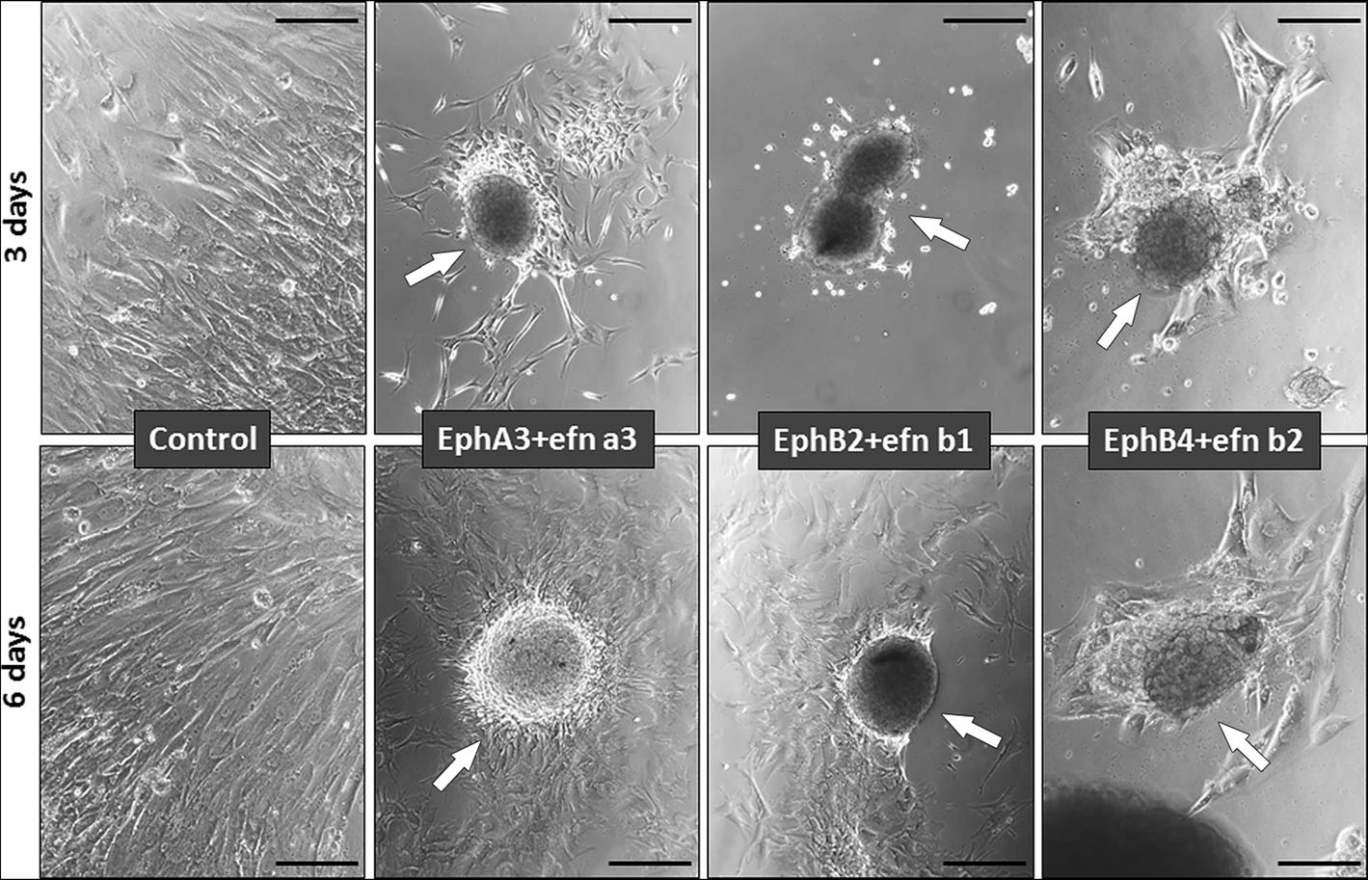
Clustered Eph/Ephrin-Fc fusion protein treatment results in cell detachment and aggregation of BM-MSC. The figure is representative of cultures supporting different treatments. Note the more or less developed cell aggregates (arrows) in treated cell cultures. Scale bars = 200 μm

**Fig. 8:**
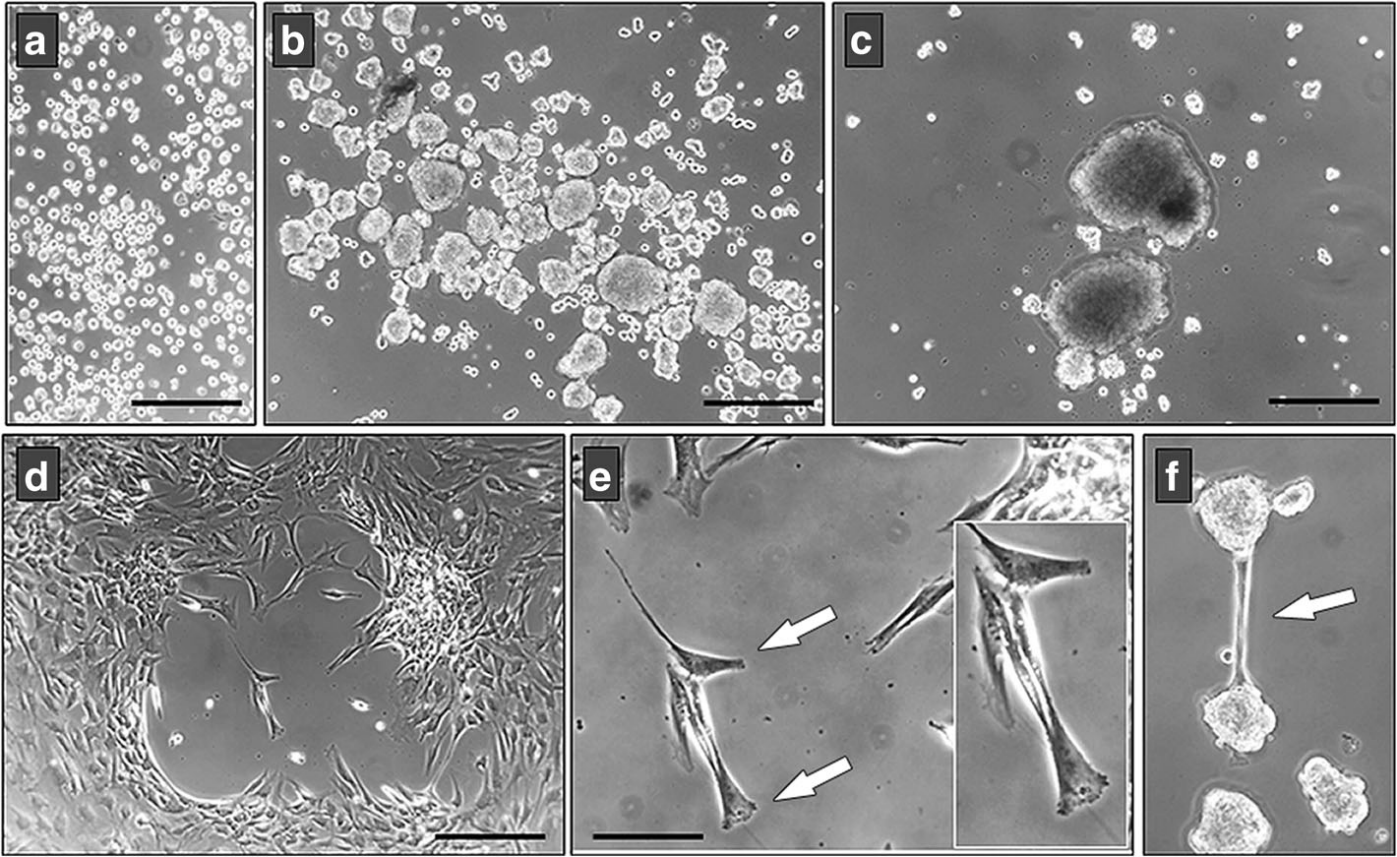
Effects of the clustered fusion protein-mediated activation of Eph/Ephrin signaling on BM-MSC morphology. **a–c** The formation of MSC aggregates in treated cultures begins with the progressive coalescence of nonadhered cells (**a**) that initially form small cellular groups (**b**) to finally group in cell masses (**c**). In other areas of cultures, treatments generate empty areas (**d**) due to retraction of cell processes (**e**, arrows). Sometimes, cell masses form “bridges” to connect each to other (**f**, arrow). Scale bars = 200 μm (**a–d**) and 100 μm (**e**)

Blockade of Eph/Ephrin signaling with soluble Eph-Fc and/or Ephrin-Fc (i.e., EphrinA4, EphrinB1, EphB4, EphB2 plus EphrinB1), which correlated with increased proportions of apoptotic BM-MSC, did not induce morphological changes in the treated BM-MSC which remained structurally similar to those of control, nontreated cultures (Fig. 6). In contrast, those cultures in which BM-MSC were activated with different combinations of clustered Eph-Fc plus Ephrin-Fc, which induced increased apoptotic BM-MSC, underwent profound changes as evidenced by the appearance of big groups of cells that apparently coalesced in cellular masses after detachment from the dish bottom (Fig. 7). Apparently the transformation of cultures began with the formation of numerous small groups of cells that gradually converged into bigger cell masses (Fig. 8a–c). In other areas, the cultures showed empty areas due to cell retraction (Fig. 8d). The treatment also induced morphological changes in individual MSC that lost their long cell processes (Fig. 8e) or occasionally constituted “bridges” connecting masses of grouped cells (Fig. 8f).

**Fig. 9:**
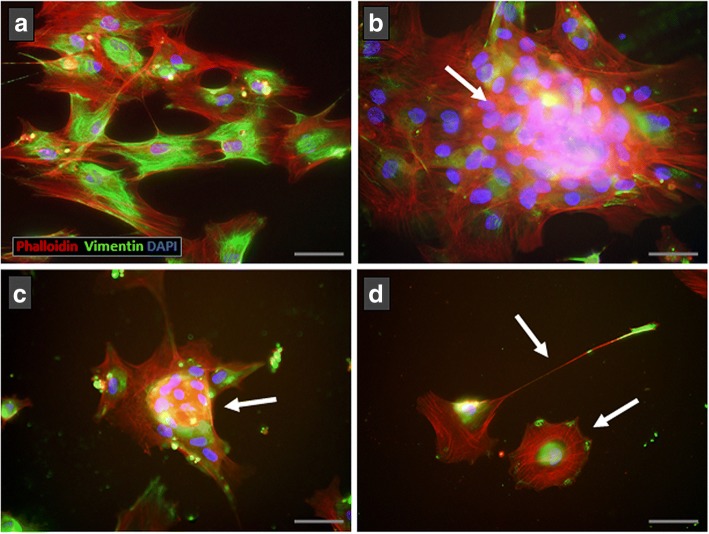
MSC cytoskeleton changes after treatment with clustered Eph/Ephrin-Fc fusion proteins. **a** Control MSC spread well on the plate showing a wide actin (red) and vimentin (green) network throughout the cell cytoplasm. In contrast, clustered Eph/Ephrin-Fc treated cells that form cell masses with numerous nuclei (blue) (**b**, **c**, arrows) exhibit a less expanded cytoskeletal network. Remarkably, isolated treated MSC (**d**) show profound morphological alterations with small clusters of vimentin in the tips of cell processes or attached areas to plate in rounded cells (arrows). Scale bars = 50 μm

These morphological changes observed in the treated BM-MSC cultures were confirmed by analyzing the organization of the cell cytoskeleton. Firstly, we compared the expression of both vimentin intermediate filaments and actin microfilaments in control, Fc-treated MSC, and activated MSC by treatment with fusion proteins (i.e., EphA3 plus EphrinA3, EphB2 plus EphrinB1, and EphB4 plus EphrinB2) that detached the cells from the culture dishes and eventually provoked their death. Whereas Fc-treated cultured cells appeared expanded, were well adhered to the substrate, and formed more or less compact cell groups with few empty areas (Fig. 9a), cell masses containing numerous nuclei constituted isolated groups in the cultures supplied with clustered fusion proteins (Fig. 9b, c). At the cellular level, Fc-treated MSC exhibited a vimentin-positive network throughout the cell cytoskeleton with abundant actin filaments from the nucleus to the periphery (Fig. 9a). In contrast, cultures receiving clustered fusion proteins contained isolated MSC with profound morphological modifications, including a rounded shape and long, narrow cell processes (Fig. 9d) and, more importantly, a particular organization of the actin network that appeared to form a compact ring surrounding the nucleus and small spots of vimentin in the cell periphery or in the tips of long cell processes (Fig. 9d).

**Fig. 10:**
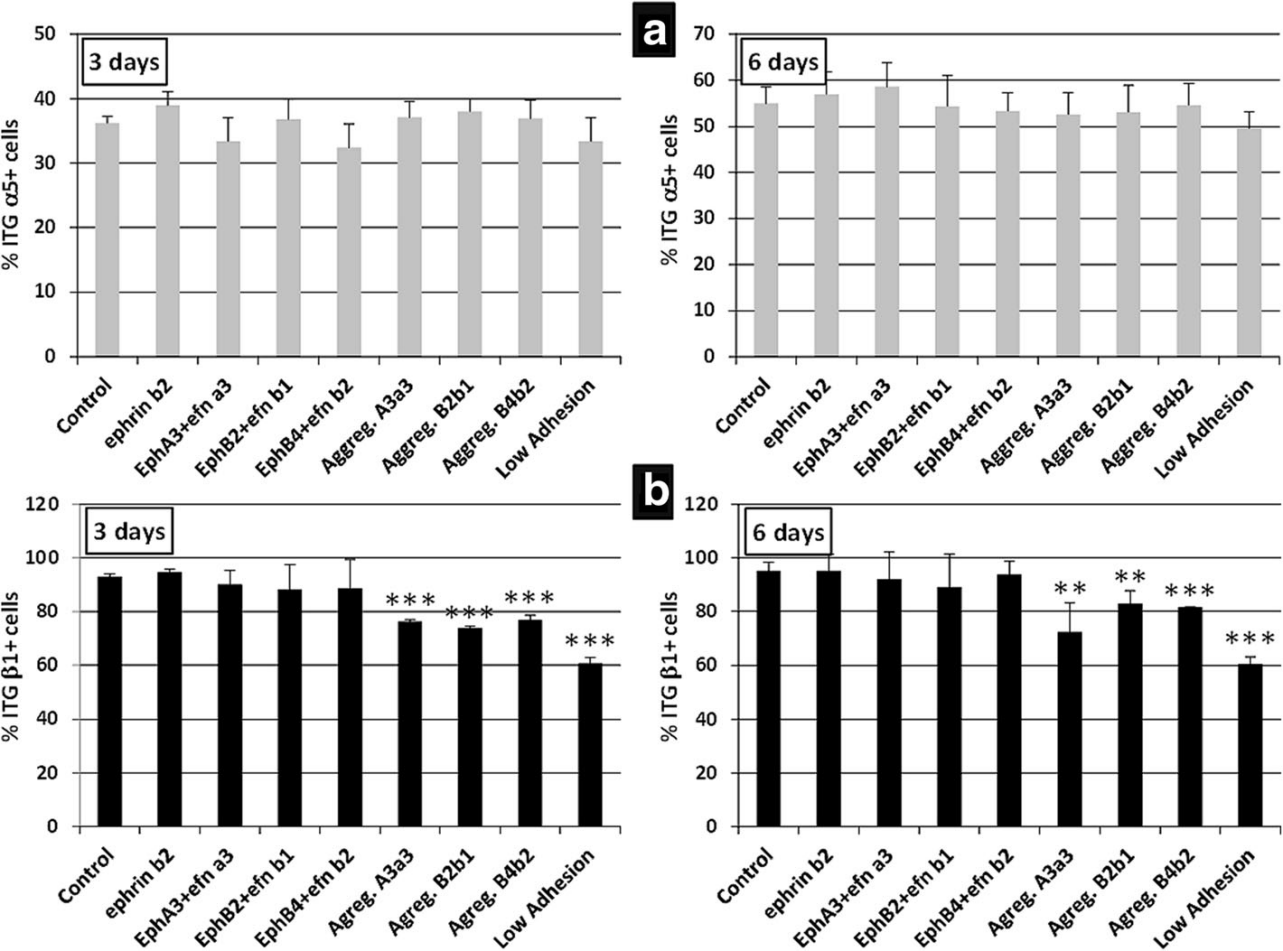
Integrin β1 chain expression is lower in BM-MSC cultures containing cell aggregates. Integrin α5 (**a**) chain and β1 (**b**) chain expression was analyzed in clustered Eph/Ephrin-Fc treated cultures that formed or did not form cell aggregates. There are no significant differences in the percentages of α5^+^ cells at any time point, but the values of β1^+^ cells are significantly lower at 3 and 6 days in cultures containing cell aggregates than in those without them. Data were obtained from five different donors. **p ≤ 0.01, ***p ≤ 0.005, versus respective control

We extended these results with a flow cytometry analysis of the proportions of BM-MSC that expressed α5 or β1 integrin chains, known to be major components of focal adhesions that maintain adhered cultured cells to surfaces coated with extracellular matrix components, largely fibronectin [34]. Whereas no changes were found in the proportions of integrin α5-positive MSC 3 and 6 days after stimulation with different combinations of fusion proteins (Fig. 10a), cultures treated with fusion proteins, some of which induced detachment of cultured cells, such as EphA3 plus EphrinA3, EphB2 plus EphrinB1, or EphB4 plus EphrinB2, or kept on low adhesion plates showed reduced proportions of integrin β1-positive MSC (Fig. 10b).

**Fig. 11:**
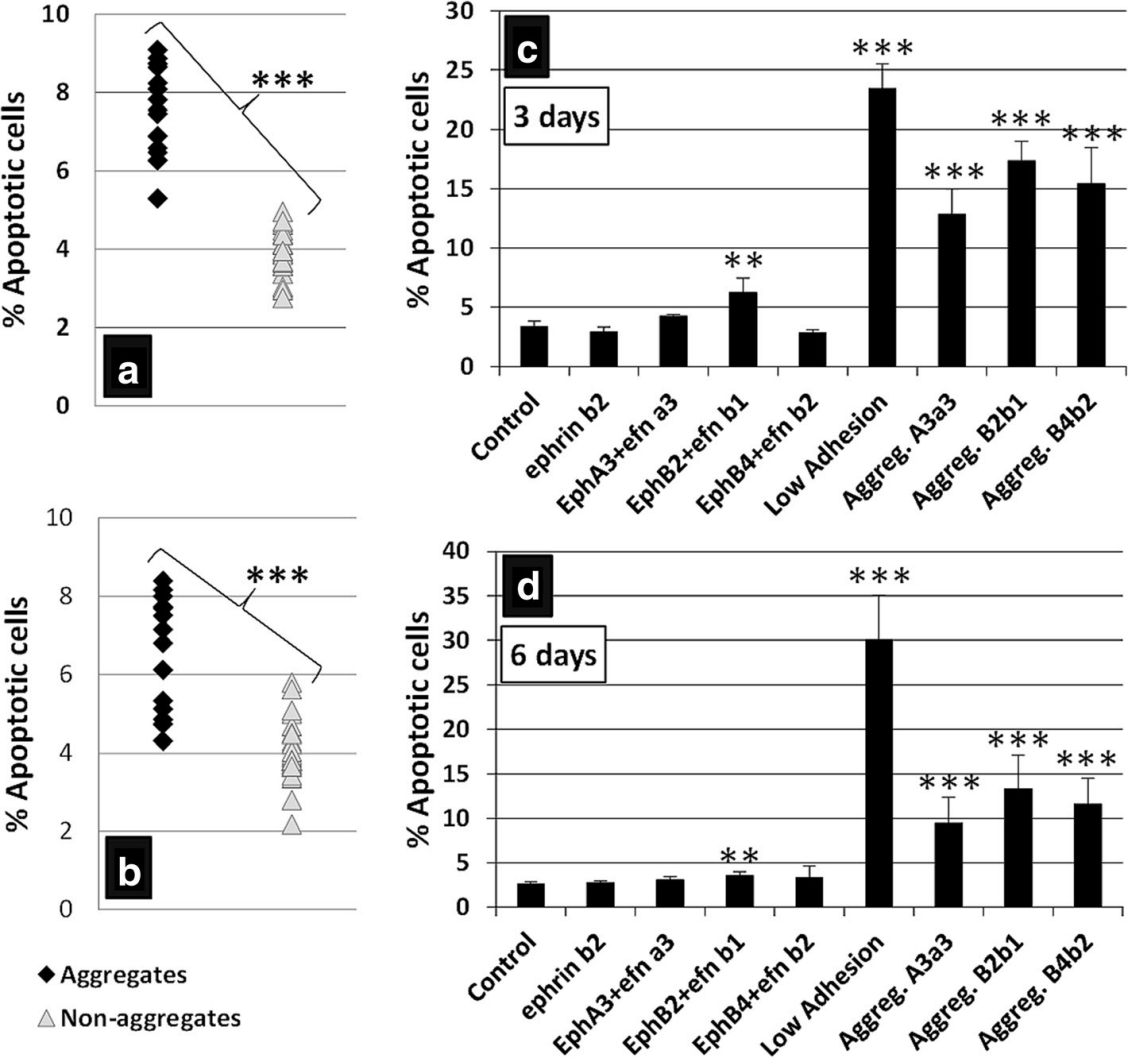
Higher percentages of apoptosis in cell aggregates than in isolated MSC from activated BM-MSC cultures. **a,b** BM-MSC cultures, treated with clustered Eph/Ephrin-Fc, which form cell aggregates (black) contain higher proportions of apoptotic cells at 3 (**a**) and 6 days of culture (**b**) than those (gray) without masses of MSC. In addition, higher proportions of apoptotic MSC occur after 3 (**c**) and 6 days (**d**) in treated cultures containing cell aggregates, in which the viability of cell masses and that of isolated MSC are evaluated independently. As a control to establish possible correlations between cell adhesion and survival, cells were also cultured on low-adhesion plates in these experiments. Data were obtained from five different donors. *p ≤ 0.05, **p ≤ 0.01, ***p ≤ 0.005, versus respective control

To further check presumptive correlations between the detachment of cultured cells and increased apoptosis observed after activation of Eph/Ephrin signaling, we firstly compared the proportions of apoptotic MSC in treated cultures that contained big cell masses with those of cultures that did not organize such cellular structures. Independently of the received treatment, cultures in which detached cells grouped in big masses showed significantly larger proportions of apoptotic MSC than those deprived of cell aggregates (Fig. 11a, b), suggesting that detachment of BM-MSC in treated cultures accounted for the increased apoptosis observed after combined treatment with some Eph-Fc and Ephrin-Fc fusion proteins. Furthermore, within the same treated cultures we comparatively analyzed the proportions of apoptotic MSC between those cells derived from the big cell aggregates and the nonclustered cells that adhered to the plate, observing higher values in the first ones (Fig. 11c, d). Supporting these results, a high percentage of apoptotic cells also occurred in the ultra-low adhesion cultures (Fig. 11c, d).

## Discussion

In the present study, we first comparatively analyzed the expression of Eph tyrosine kinase receptors and their ligands, Ephrins, in both human BM-MSC and Ad-MSC. Isolation and immunophenotyping characterization of MSC derived from the two tissues were similar, and we did not find differences in the expression of cell markers classically used for defining MSC (i.e., CD14, CD34, CD45, CD73, CD90, CD105, CD166, HLA-DR). However, other molecules are differentially expressed in the two cell types. It has been reported that CD49d is expressed only in Ad-MSC whereas CD106 occurs only in BM-MSC [35, 36]. In addition, several chemokine receptors important for MSC mobilization are expressed more in Ad-MSC than in BM-MSC [37]. In fact, our current study demonstrates that Eph/Ephrin transcripts are more abundant in BM-MSC than in Ad-MSC, especially those for EphA3, A7, and B2, and EphrinA1, A3, and B2. In agreement with this, expression of several Eph and Ephrins, belonging to family B rather than to family A, and especially EphB2, EphB4, EphrinB1, and EphrinB2, have been reported to be expressed in MSC isolated from bone marrow, cord blood, and bone [6, 8, 10, 13, 38, 39]. These molecules appear to be relevant for bone marrow physiology. Thus, the EphB2/EphrinB2 pair has been reported to be critical in regulating hematopoietic progenitor cell function [9, 14] and exit from the bone marrow [13, 40], and Eph/Ephrin signaling promotes MSC migration and recruitment into injury sites [6, 19].

We also wanted to verify the effects of either blockade or activation of Eph/Ephrin signaling on in-vitro expansion, survival, and proliferation of MSC. As known already, it is difficult to expand ex vivo fresh BM-MSC, making interesting any procedure focused on obtaining enough cells as a first step for its further use as a therapeutic agent. Distinct combinations of soluble, dimeric Eph/Ephrin fusion proteins, particularly EphB4-Fc, EphrinA4-Fc, EphA3-Fc plus EphrinA3-Fc, and EphB2-Fc plus EphrinB1-Fc, significantly decrease the number of MSC. Remarkably, most of these molecules affecting MSC growth are strongly expressed in BM-MSC, as shown by qRT-PCR. On the other hand, this decreased cellularity observed in treated MSC cultures correlates with significantly increased proportions of apoptotic cells that appear on day 3 of culture, but not with changes in the percentages of cycling cells.

Unfortunately, there are no data on this topic related to MSC, and results are contradictory in other cell systems. Eph stimulation in different cell types frequently correlates with increased cell proliferation [41–43], but inhibits it in other cases [4, 44]. Other studies report no changes in the percentage of proliferating cells after different treatments with fusion proteins [45] or in the absence of distinct Eph [46]. Our current results also show no changes in the proportions of dividing BM-MSC, except for the significant decreased proportions of cycling BM-MSC observed after 3 days of culture with activating clustered EphA3-Fc plus EphrinA3-Fc, EphB2-Fc plus EphrinB1-Fc, and EphB4-Fc plus EphrinB2-Fc fusion proteins.

On the other hand, our results demonstrate that BM-MSC survival is particularly sensitive to the blockade of signaling pathways mediated by Eph and/or Ephrins highly expressed in BM-MSC. In general, the blockade of Eph/Ephrin interactions mediated by soluble, dimeric fusion proteins correlates with decreased cell content and increased proportions of apoptotic cells [30, 47] as we found, and mice deficient in different Eph (i.e., EphA4, EphB2, EphB3) or EphrinA3 exhibit high levels of apoptotic cells [41, 48–50]. However, EphrinB2-Fc treatment results in growth inhibition in human umbilical vein endothelial cells (HUVEC), but in growth promotion in MCF-7 cells [51].

It is difficult to explain how Eph and Ephrins function. Different results support that the addition of soluble, dimeric Eph-Fc proteins block Eph/Ephrin interactions but could also stimulate Ephrin-dependent reverse signaling; similarly, Ephrin-Fc fusion proteins would activate forward signals mediated through Eph [29, 30, 52]. Although soluble, dimeric Eph-Fc and, especially, Ephrin-Fc fusion proteins have low efficiency in inducing activating signals [53], it has been reported that soluble EphrinB-Fc proteins activate EphB4 [54], and monomeric EphrinA4-Fc is able to induce biological responses [55]. On the other hand, whereas dimeric or monomeric EphrinA2 activates EphA [29], EphrinB1 needs to be clustered repeatedly to activate EphB2 [56].

Accordingly, we wished to confirm any relationships between the blockade of Eph/Ephrin signaling by soluble dimeric fusion proteins and increased proportions of apoptotic BM-MSC using other previously tested Eph/Ephrin blockers. Uni-PR129 is a conjugate of lithocholic acid with l-homotryptophan that is able to block in vitro angiogenesis in HUVEC by inhibiting both EphA2 and EphB4 activity at micromolar concentrations [57], but also those mediated by EphrinB1 and EphB [32]. On the other hand, Uni-PR500 is a new reversible and competitive antagonist able to inhibit EphA5-EphrinA5 binding [58]. By using either of these two blockers, the proportions of apoptotic BM-MSC increased at both 3 and 6 days of culture. A similar condition is observed when MSC receive a mAb B11 that binds EphrinB2 [31]. All these results indicate that increased proportions of apoptotic BM-MSC correlate with the blockade of Eph/Ephrin signaling mediated by soluble fusion proteins rather than with its activation.

Independently of this controversy, it is generally assumed that clustered Eph or Ephrins activate Eph/Ephrin signaling [33] and decrease cell apoptosis [45, 59, 60]. However, it has been reported that EphB6 cross-linking induces apoptosis of Jurkat cells [61], and both EphB2-Fc and EphrinB1-Fc immobilized proteins modulate anti-CD3 Ab-induced apoptosis [41]. It has been proposed that these opposite results reflect a specific response of the cell type and is dependent on clustered protein concentration [41].

Our current results on BM-MSC treated with clustered Eph/Ephrin fusion proteins are remarkable for two reasons. Firstly, some molecules (i.e., EphrinA4, EphrinA3, EphB2, EphB4, EphrinB1, and EphrinB2) increase apoptosis when supplied alone as soluble blockers, but do not affect BM-MSC survival when activating Eph/Ephrin signaling pathways. On the contrary, combinations of clustered Eph-Fc plus Ephrin-Fc proteins induce increased proportions of apoptotic BM-MSC. On the other hand, activation of BM-MSC by treatment with clustered individual Eph-Fc or Ephrin-Fc fusion proteins or blockade of Eph/Ephrin interactions by soluble proteins do not provoke changes in MSC morphology. However, the treatment with clustered Eph-Fc plus Ephrin-Fc proteins induces notable changes in the morphology of BM-MSC that result in cell detachment. Although we do not know the underlying reasons for this behavior, it is evident that the combined activation of Eph and Ephrin signals generates important alterations in the biology of BM-MSC that do not occur when cells are independently stimulated by either Eph-Fc or Ephrin-Fc alone. As indicated, each Eph receptor can bind several Ephrins and vice versa, signaling bidirectionally through Eph (forward signal) or Ephrin (reverse signal) [5]. Thus, a well-regulated balance of forward and reverse signals is necessary for mediating attraction-repulsion fine-tuning cell interactions [62]. In this respect, we have previously observed different responses when the Eph/Ephrin signaling was unbalanced, comparing the thymic phenotype of EphB2KO- and EphB2LacZ-deficient mice in terms of thymic epithelial cell (TEC) survival [48], formation of thymocyte-TEC conjugates [62], and in-vitro attraction of lymphoid progenitors into the thymus [63]. EphB2LacZ mice express a truncated form of EphB2 unable to transmit a forward signal, but it stimulates a reverse one [64]. In addition, unbalanced Eph/Ephrin signaling in chimeric SCID mice receiving bone marrow lymphoid progenitor cells from different EphB-deficient mice results in impaired T-cell differentiation [62]. Similar results are seen when EphrinB1 or EphrinB2 are specifically deleted in thymocytes or TEC [65]. Therefore, different combinations of Eph and Ephrins signals cause different phenotypes, as now observed in the behavior of BM-MSC. On the other hand, results on the effects of Eph/Ephrin on cell attachment are controversial, and even the same molecule can promote cell repulsion or cell adhesion according to the cell type [4, 66, 67]. In numerous examples, Eph forward signaling results in cell repulsion [44, 68], although adhesive responses have also been reported [14, 69].

In agreement with our current results, some studies using BM-MSC indicate that Eph activation correlates with morphological changes and cell death. Arthur and colleagues [6] reported that Stro-1^+^ BM-MSC treated for 3 h with either EphB2-Fc or EphB4-Fc, but not with EphrinB1-Fc or EphrinB2-Fc proteins, underwent roundness and decreased size. Furthermore, EphA3^+^ CD29^+^ Sca-1^hi^ MSC respond to an EphA3-activating mAb by fast contraction and apoptosis [20]. Rounded cells occur in our BM-MSC cultures activated by clustered Eph-Fc plus Ephrin-Fc fusion proteins. Other cells that are detached from the culture dish, converge in big cell masses and eventually die. To our knowledge, similar findings have not been previously described, although there is abundant evidence to support that Eph signaling correlates with profound changes in the cytoskeleton and focal adhesions, also demonstrated herein, which results in changes in cell shape and/or cell detachment and migration [4]. On the other hand, it has been reported that cells plated on substrates with different topographies and rigidities exhibit different behaviors in terms of proliferation, differentiation, and apoptosis [70].

Thus, the unexpected increased apoptosis observed in the clustered Eph-Fc plus Ephrin-Fc treated BM-MSC cultures could be related to the cell detachment and accumulation of big cell masses. Two results in this study support this hypothesis. Firstly, cultures containing detached big cell masses, independently of performed treatment, exhibit significantly higher proportions of apoptotic cells than those without detached cell groups. Furthermore, when big cell masses are isolated from the cultures and their proportions of apoptotic cells evaluated versus those of other areas of the same cultures that do not form big cell masses, the proportions of apoptotic cells are significantly larger in the first case.

On the other hand, MSC “sense” the underlying rigidity of the extracellular matrix as part of their differentiation into various cell types [71], and have been shown to respond to the topographical constraints of their attachment to the extracellular matrix to differentiate into osteogenic or adipogenic lineages [72]. As is known, MSC differentiate to distinct mesoderm cell lineages, particularly to bone lineage cells and adipocytes, when adequate culture conditions are provided. In addition, it is generally accepted that factors favoring osteogenesis inhibit adipogenesis and vice versa [73, 74]. In all experimental conditions, BM-MSC produce bone cells at similar levels to those of control, Fc-treated cultures, except in MSC cultures treated with EphA3-Fc plus EphrinA3-Fc, which show decreased proportions of ALP per microgram of total protein (Additional file 1: Figure S1). Other authors have pointed out important differences in the osteogenic capacities of MSC after activation by EphB4-Fc [75]. EphB2-Fc treatment increases osteogenesis, and blocking EphB1-Fc or EphB1-Fc or EphB4-Fc signal peptides inhibits it [6]. On the other hand, EphA5 has been proposed as an inhibitor of the osteogenic differentiation of BM stromal cells [23]. It is difficult to explain these different results that, as with other properties of these molecules, are dependent on the studied cell types, experimental approaches, and/or systems for evaluating cell differentiation.

## Conclusions

In summary, our results confirm the pattern of expression of Eph and Ephrins in in-vitro expanded MSC described by other authors, and demonstrate the relevance of these molecules for the survival of BM-MSC. We pay attention particularly to the finding that some combinations of clustered Eph-Fc and Ephrin-Fc fusion proteins, used frequently to activate Eph/Ephrin signaling, profoundly affect the attachment of BM-MSC to the substrate, favoring their cell death. Whether this condition also occurs in vivo, affecting the reported effects of Eph and Ephrins on the biology of hematopoietic progenitor cells and stromal BM cells, remains to be verified.

## Additional file

Additional file 1: **Figure S1.** Activation of BM-MSC with clustered EphA3 plus EphrinA3-Fc fusion proteins induces low production of ALP. Percentage of ALP per milligram total protein in lysates of BM-MSC activated with several clustered Eph plus Ephrin-Fc fusion proteins and 7 days of treatment with osteogenic differentiation medium. Note the reduced ALP values in MSC cultures treated with EphA3 plus EphrinA3-Fc proteins. The significance of values with reference to the control group (DMEM) is indicated as **p ≤ 0.01. (JPG 74 kb)

## Abbreviations

Ad-MSC: Adipose tissue-derived mesenchymal stromal cells
ALP: Alkaline phosphatase
BM-MSC: Bone marrow-derived mesenchymal stromal cells
DMEM: Dulbecco’s modified Eagle’s medium
DMSO: Dimethyl sulfoxide
FBS: Fetal bovine serum
HSPC: Hematopoietic stem precursor cell
HUVEC: Human umbilical vein endothelial cells
mAb: Monoclonal antibody
MSC: Mesenchymal stromal cells
OD: Optical density
PBS: Phosphate-buffered saline
PI: Propidium iodide
qRT-PCR: Quantitative real-time polymerase chain reaction
TEC: Thymic epithelial cells
