# Supplementary figures and images for: Eph/Ephrin-mediated stimulation of human bone marrow mesenchymal stromal cells correlates with changes in cell adherence and increased cell death
Source: Stem Cell Res Ther. 2018 Jun 26;9:172. doi: 10.1186/s13287-018-0912-3 (PMC6019728; doi:10.1186/s13287-018-0912-3)

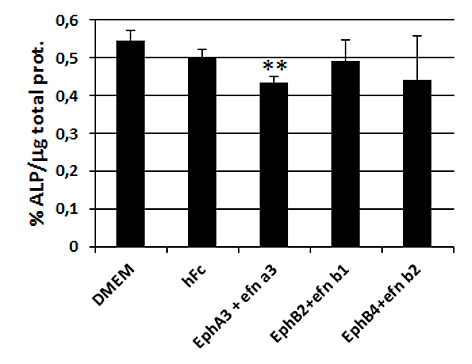

Supplement: Supplementary file 1 — Figure S1. Activation of BM-MSC with clustered EphA3 plus EphrinA3-Fc fusion proteins induces low production of ALP. Percentage of ALP per milligram total protein in lysates of BM-MSC activated with several clustered Eph plus Ephrin-Fc fusion proteins and 7 days of treatment with osteogenic differentiation medium. Note the reduced ALP values in MSC cultures treated with EphA3 plus EphrinA3-Fc proteins. The significance of values with reference to the control group (DMEM) is indicated as **p ≤ 0.01. (JPG 74 kb) [file 13287_2018_912_MOESM1_ESM.jpg]
